# *Agerinia marandati* sp. nov., a new early Eocene primate from the Iberian Peninsula, sheds new light on the evolution of the genus *Agerinia*

**DOI:** 10.7717/peerj.3239

**Published:** 2017-04-27

**Authors:** Joan Femenias-Gual, Raef Minwer-Barakat, Judit Marigó, Miquel Poyatos-Moré, Salvador Moyà-Solà

**Affiliations:** 1 Institut Català de Paleontologia Miquel Crusafont, Universitat Autònoma de Barcelona, Cerdanyola del Vallès, Barcelona, Spain; 2 Centre de Recherches sur la Paléobiodiversité et les Paléoenvironnements (CR2P, UMR 7207), Sorbonne Universités –MNHN, CNRS, UMPC-Paris6–, Muséum National d’Histoire Naturelle, Paris, France; 3 Department of Geosciences, University of Oslo, Sem Sælands vei 1, Oslo, Norway; 4 Unit of Anthropology, BABVE Department, Universitat Autònoma de Barcelona, Cerdanyola del Vallès, Barcelona, Spain; 5 ICREA, Pg. Lluís Companys 23, Barcelona, Spain

**Keywords:** Adapiformes, Notharctidae, Paleogene, Spain

## Abstract

**Background:**

The Eocene was the warmest epoch of the Cenozoic and recorded the appearance of several orders of modern mammals, including the first occurrence of Euprimates. During the Eocene, Euprimates were mainly represented by two groups, adapiforms and omomyiforms, which reached great abundance and diversity in the Northern Hemisphere. Despite this relative abundance, the record of early Eocene primates from the European continent is still scarce and poorly known, preventing the observation of clear morphological trends in the evolution of the group and the establishment of phylogenetic relationships among different lineages. However, knowledge about the early Eocene primates from the Iberian Peninsula has been recently increased through the description of new material of the genus *Agerinia* from several fossil sites from Northeastern Spain.

**Methods:**

Here we present the first detailed study of the euprimate material from the locality of Masia de l’Hereuet (early Eocene, NE Spain). The described remains consist of one fragment of mandible and 15 isolated teeth. This work provides detailed descriptions, accurate measurements, high-resolution figures and thorough comparisons with other species of *Agerinia* as well with other Eurasian notharctids. Furthermore, the position of the different species of *Agerinia* has been tested with two phylogenetic analyses.

**Results:**

The new material from Masia de l’Hereuet shows several traits that were previously unknown for the genus *Agerinia,* such as the morphology of the upper and lower fourth deciduous premolars and the P_2_, and the unfused mandible. Moreover, this material clearly differs from the other described species of *Agerinia*, *A. roselli* and *A. smithorum*, thus allowing the erection of the new species *Agerinia marandati*. The phylogenetic analyses place the three species of *Agerinia* in a single clade, in which *A. smithorum* is the most primitive species of this genus.

**Discussion:**

The morphology of the upper molars reinforces the distinction of *Agerinia* from other notharctids like *Periconodon*. The analysis of the three described species of the genus, *A. smithorum, A. marandati* and *A. roselli*, reveals a progressive change in several morphological traits such as the number of roots and the position of the P_1_ and P_2_, the molarization of the P_4_, the reduction of the paraconid on the lower molars and the displacement of the mental foramina. These gradual modifications allow for the interpretation that these three species, described from the early Eocene of the Iberian Peninsula, are part of a single evolutionary lineage. The stratigraphical position of Masia de l’Hereuet and Casa Retjo-1 (type locality of *A. smithorum*) and the phylogenetic analyses developed in this work support this hypothesis.

## Introduction

One of the most important steps in the early radiation of the primate clade was the appearance and diversification of Euprimates, also known as true primates or primates of “modern aspect” (Bloch et al., 2007; Silcox et al., 2015). Within Euprimates, two main groups were differentiated in the early Eocene, Omomyiformes and Adapiformes, which may be related to the main clades of living primates (haplorhines and strepsirrhines, respectively) following the more accepted theory (e.g., Seiffert et al., 2009; Ni et al., 2013; Godinot, 2015). However, several researchers consider Adapiformes as the stem group of the clade Haplorhini (e.g., Gingerich et al., 2010; Gingerich, 2012).

The first records of these groups in Europe correspond to the omomyiforms *Teilhardina, Melaneremia* and *Nannopithex* and the adapiforms *Donrussellia*, *Cantius, Protoadapis* and *Agerinia* (Gebo, 2002; Godinot, 2015). Despite this relative diversity, the early Eocene primate record is still scarce and poorly known, preventing the establishment of clear phylogenetic relationships among known taxa. As the most common elements found in the fossil record are teeth, changes observed in dental morphology are the primary basis for distinguishing evolutionary lineages.

Recent works dealing with European Eocene primates have focused on the description of new material (Hooker, 2007; Hooker, 2012; Hooker & Harrison, 2008; Marigó, Minwer-Barakat & Moyà-Solà, 2011; Marigó, Minwer-Barakat & Moyà-Solà, 2013; Gebo, Smith & Dagosto, 2012; Gebo et al., 2015; Minwer-Barakat, Marigó & Moyà-Solà, 2012; Minwer-Barakat et al., 2013; Femenias-Gual et al., 2015), the revision of previous taxonomic assignations (Minwer-Barakat, Marigó & Moyà-Solà, 2013; Minwer-Barakat, Marigó & Moyà-Solà, 2016; Marigó et al., 2014) and the establishment of relationships between different taxa (Smith, Rose & Gingerich, 2006; Marigó, Minwer-Barakat & Moyà-Solà, 2010; Marigó, Minwer-Barakat & Moyà-Solà, 2013; Minwer-Barakat et al., 2017), with some exceptions focused on the diet (Ramdarshan, Merceron & Marivaux, 2012), the locomotor behaviour (Marigó et al., 2016) and the endocranial anatomy (Ramdarshan & Orliac, 2016) of several species. However, only a few contributions have been published regarding European primates from the early Eocene, recently including the revision of *Agerinia roselli* from Les Saleres and the description of the new species *Agerinia smithorum* from Casa Retjo-1 (Femenias-Gual et al., 2016a and Femenias-Gual et al., 2016b, respectively). The former work allowed for the identification of several traits of *A. roselli* that were not described previously, such as the presence of two roots situated mesially with respect to the P_3_ or the presence of a tiny paraconid on the M_1_. On the other hand, *A. smithorum* is characterized by the presence of a two-rooted P_2_, a well-developed paraconid on the M_1_ and a tiny one on the M_2_, among other features. Based on these primitive traits, these authors proposed *A. smithorum* as a probable ancestor of *A. roselli*.

Here we present the first detailed study of new euprimate material found in the locality of Masia de l’Hereuet (early Eocene, NE Spain), where the presence of the plesiadapiform *Arcius* was already noted by Marigó et al. (2012). A preliminary study of this material was made by Femenias-Gual et al. (2014), who did not give a taxonomic determination. In the present work, after comparison with the material of *A. roselli* from Les Saleres and *A. smithorum* from Casa Retjo-1, all the euprimate teeth found from Masia de l’Hereuet can be confidently assigned to the genus *Agerinia*. Moreover, some morphological traits different from those of *A. roselli* and *A. smithorum* allow the erection of a new species. The material from Masia de l’Hereuet allows the first description of the deciduous upper and lower teeth of *Agerinia*; in addition, this sample includes several upper molars, which were still unknown for *A. roselli* and *A. smithorum*, and only known for some small samples of *Agerinia* sp. such as those from Casa Ramón and Condé-en-Brie (Peláez-Campomanes, 1995; Herbomel & Godinot, 2011).

## Geological setting

**Figure 1:**
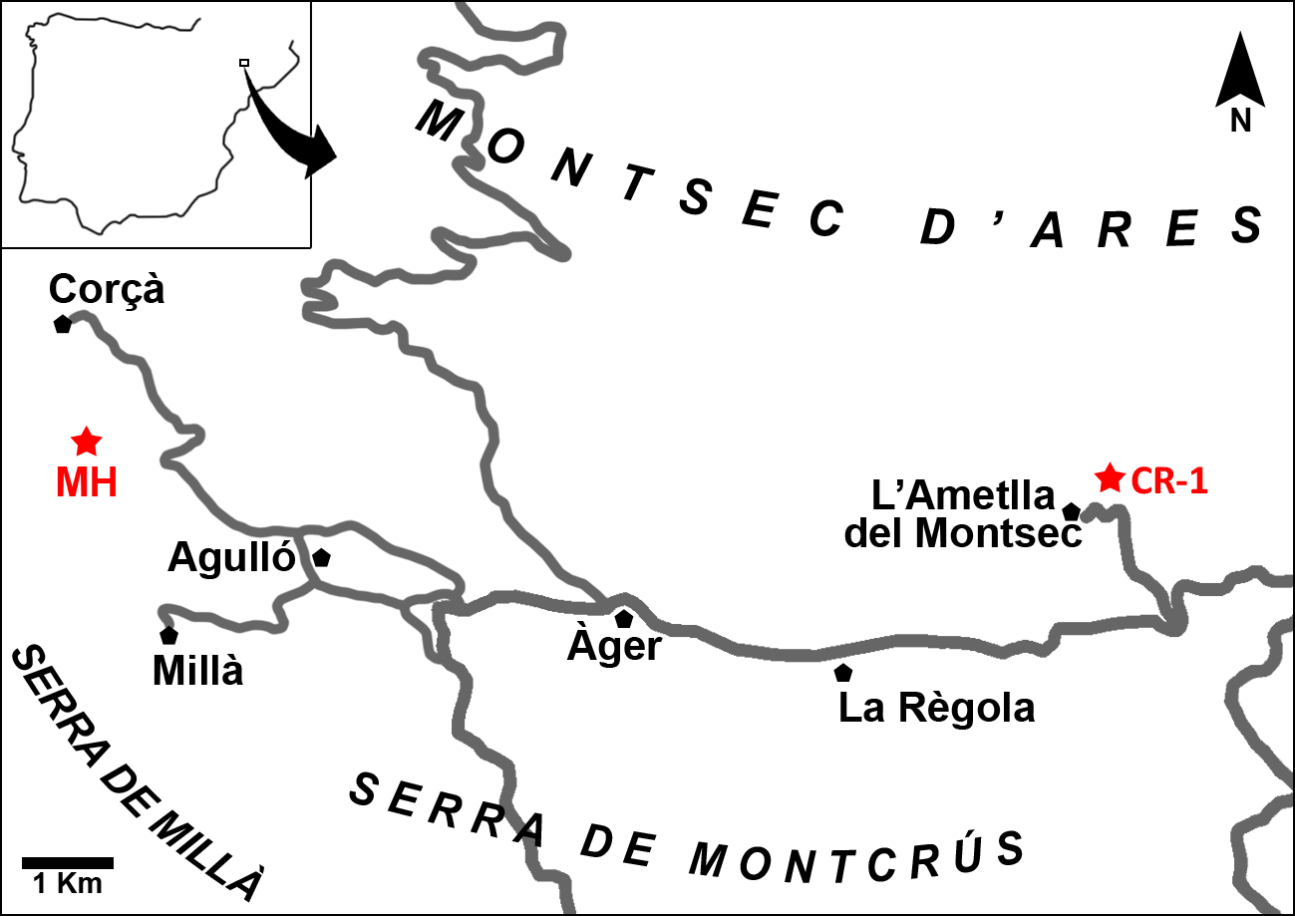
Map of the Àger sub-basin (Southern Pyrenean Basins, NE Spain). Red stars represent the placement of the early Eocene fossil sites Masia de l’Hereuet (MH) and Casa Retjo-1 (CR-1). Modified from Femenias-Gual et al. (2016b).

The fossil site of Masia de l’Hereuet is located to the south of the path that connects the villages of Corçà and Agulló (Fig. 1), in the western sector of the Àger valley (Lleida province, NE Spain). Geologically, this locality is situated in the continental Eocene deposits of the Corçà formation in the Àger sub-basin, included in the south Pyrenean foreland basin (Puigdefàbregas et al., 1989). The continental deposits of the Corçà Formation overlie early Eocene transitional deltaic deposits of the Ametlla Formation (Mutti et al., 1985; Dreyer & Fält, 1993; Zamorano, 1993), and are mainly made up of different terrigenous deposits, including clays and sandstones interbedded with some conglomeratic levels. Fine grained deposits (mainly clays) are interpreted as floodplain deposits, while sandstones and conglomerates are related to complex multi-storey stacking of braided and meandering river channels (Crusafont-Pairó & Rosell-Sanuy, 1966; Solé, 1985; Checa, 1995; Poyatos-Moré et al., 2013).

**Figure 2:**
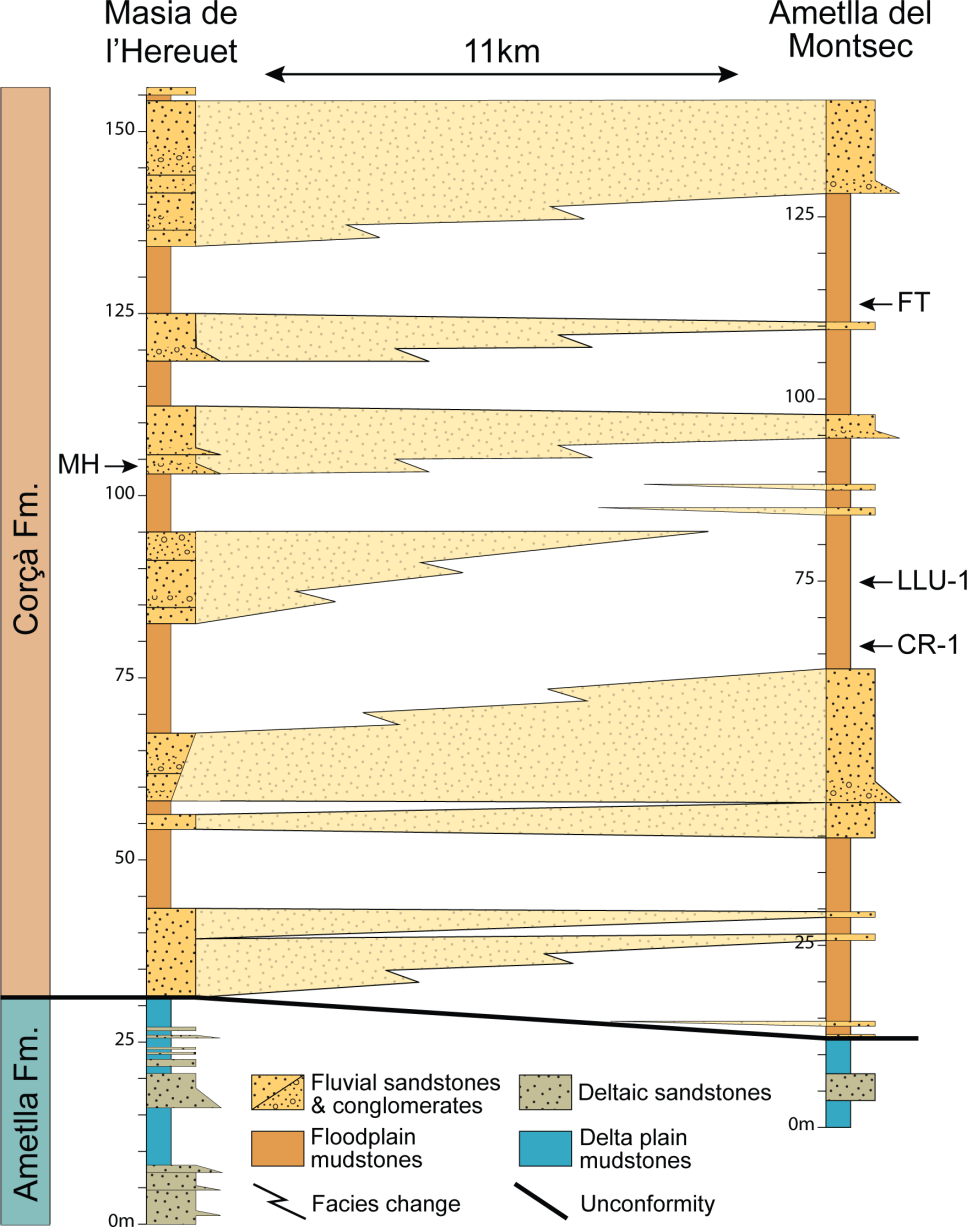
Regional correlation between the sections of Masia the l’Hereuet and L’Ametlla del Montsec. The scheme shows the thickness and lateral facies changes of the main sandstone units and the location of the fossil sites: Masia de l’Hereuet (MH), Font del Torricó (FT), Casa Llúcio-1 (LLU-1) and Casa Retjo-1 (CR-1).

Several fossil-bearing levels have been identified within the deposits of the Corçà Formation in the Àger sub-basin, including the locality of Casa Retjo-1, type locality of the species *Agerinia smithorum.* Two representative sedimentary logs were measured in the sections of Masia de l’Hereuet and L’Ametlla del Montsec (where Casa Retjo-1 is located), separated by approximately 11 km (Fig. 1). Their stratigraphic correlation is shown in Fig. 2. In both sections, the lithology is mainly composed of several 5–20 m-thick fluvial sand-rich units alternating with 10’s m-thick, fine-grained packages of floodplain mudstones (Corçà Fm.), and overlying transitional deltaic deposits (Ametlla Fm.). The correlation between the two studied sections allows placing the Masia de l’Hereuet fossil site stratigraphically two fluvial units above Casa Retjo-1, indicating a relative younger age for the former locality.

The mammalian fossil remains found in Masia de l’Hereuet allowed Antunes et al. (1997) to assign this site to the Grauvian (MP10, Reference Level of the mammalian biochronological scale for the European Palaeogene, Schmidt-Kittler, 1987; Aguilar, Legendre & Michaux, 1997). Later on, Badiola et al. (2009) considered that this fossil site was older than previously thought, and assigned Masia de l’Hereuet to the Neustrian (MP8+9) after a revision of the rodents, artiodactyls and perissodactyls from this locality. Recently, the lizards from Masia de l’Hereuet have also been described by Bolet (in press).

## Material and Methods

### Studied material

The material studied comes from the fossil site Masia de l’Hereuet and consists of a right mandible fragment (IPS-82807) that preserves the teeth from P_2_ to P_4_ and the root of the P_1_, and 15 isolated teeth identified as: one right and one left dP_4_ (IPS-82796; IPS-82797); one left P_4_(IPS-82806); two complete and one broken left M_1_ (IPS-82800; IPS-82801; IPS-82802); one complete left M_2_ and one fragment of a right M_2_ (IPS-82805; IPS-82798); two left M_3_ (IPS-82803, IPS-82804); one right dP^4^ (IPS-82814); one entire and one broken left M^1^ (IPS-82808; IPS-82809); one left M^2^ (IPS-82815); and one left M^3^ (IPS-82799). All this material is housed at the Institut Català de Paleontologia Miquel Crusafont, ICP (Sabadell, Spain).

### Comparative sample

The material studied from Masia de l’Hereuet has been directly compared with the specimens of *Agerinia roselli* from Les Saleres (Spain) and *Agerinia smithorum* from Casa Retjo-1 (Spain), both stored at the ICP collections. In addition, the studied sample has been compared with *Agerinia* cf. *roselli* from Azillanet (France), belonging to the collections of the Université de Montpellier; and with *Agerinia* sp. from Condé-en-Brie (France), *Donrussellia gallica, Pronycticebus gaudryi and Protoadapis curvicuspidens,* which are stored at the MNHN. It has also been compared with high-quality casts of *Periconodon huerzeleri, Donrussellia magna, Donrussellia provincialis, Europolemur klatti, Protadapis ignoratus, Cantius eppsi, Marcgodinotius indicus* and *Asiadapis cambayensis,* also stored in the MNHN. Finally, comparisons with *Agerinia* sp. from Casa Ramón (Spain), cf. *Agerinia* from Rians (France), *Periconodon* sp. from Eckfeld Maar (Germany), *Periconodon lemoinei, Periconodon jaegeri, Donrussellia lusitanica, Donrussellia russelli, Donrussellia louisi, Darwinius masillae, Europolemur koenigswaldi, Europolemur dunaifi, Europolemur kelleri, Protoadapis angustidens, Protoadapis brachyrhynchus, Protoadapis weigelti, Protoadapis muechelnensis* and *Cantius savagei*, are based on published data.

### Dental nomenclature, measurements and micrographs

The dental nomenclature used in the descriptions is that proposed by Szalay & Delson (1979). Measurements have been taken with an optic caliper “Nikon measuroscope 10” connected to a monitor “Nikon SC112”, using the criteria described by Marigó, Minwer-Barakat & Moyà-Solà (2010). Micrographs have been taken using the Environmental Scanning Electron Microscope (ESEM) at Universitat de Barcelona.

### New zoological taxonomic name

The electronic version of this article in Portable Document Format (PDF) will represent a published work according to the International Commission on Zoological Nomenclature (ICZN), and hence the new names contained in the electronic version are effectively published under that Code from the electronic edition alone. This published work and the nomenclatural acts it contains have been registered in ZooBank, the online registration system for the ICZN. The ZooBank LSIDs (*Life Science Identifiers*) can be resolved and the associated information viewed through any standard web browser by appending the LSID to the prefix http://zoobank.org/. The LSID for this publication is: urn:lsid:zoobank.org:pub:729814E7-5509-48C1-9FF0-84D3E515D909. The online version of this work is archived and available from the following digital repositories: PeerJ, PubMed Central and CLOCKSS.

### Phylogenetic analyses

Two phylogenetic analyses were run using a version of a character-taxon matrix of living and extinct primates as well as euarchontan outgroups that was originally published by Seiffert et al. (2005). This matrix has been successively modified (Seiffert et al., 2009) and a recent version was used by Marigó et al. (2016). The matrix analysed here (Data S1) includes 391 characters and 109 taxa for the first analysis, or 112 taxa for the second analysis (see ‘Results of the Phylogenetic Analyses’). The three taxa added in the matrix for the second analysis are *Donrussellia gallica, Periconodon huerzeleri* and *Darwinius masillae*. Their codification was taken from older versions of the matrix used by Seiffert et al. (2010) and Marigó, Minwer-Barakat & Moyà-Solà (2011). In both analyses some multistate characters were treated as ordered, and those with polymorphisms scored as intermediate states were scaled to a half-step so that transitions between adjacent “fixed” states in morphoclines were equal to one full step. Both parsimony analyses were run in PAUP 4.0b10 (Swofford, 1998) for 5,000 replicates with random addition sequence and the tree-bisection-reconnection branch-swapping algorithm. Both analyses were constrained by a molecular scaffold using a constraint tree (Data S2), and treated premolar loss as reversible.

## Systematic Paleontology

**Table:** 

Order PRIMATES Linnaeus, 1758
Suborder STREPSIRRHINI Geoffroy Saint-Hilaire, 1812
Infraorder ADAPIFORMES Hoffstetter, 1977
Family NOTHARCTIDAE Trouessart, 1879
Genus *AGERINIA* Crusafont-Pairó, 1973

*AGERINIA MARANDATI* sp. nov.

urn:lsid:zoobank.org:act:02E82A3B-58B0-4FB8-AE2C-64E1517A0F24

Figs. 3–5

*Derivation of name.* This species is named after Bernard Marandat (Institut des Sciences de l’Évolution, Université de Montpellier, France), in recognition of his outstanding contribution to the knowledge of Paleogene mammals.

*Holotype.* Left isolated M_1_ (IPS-82801) from Masia de l’Hereuet, stored in the Institut Català de Paleontologia Miquel Crusafont (ICP), Sabadell, Spain.

*Hypodigm.* Right mandible fragment preserving the root of P_1_ and the teeth from P_2_ to P_4_ (IPS-82807); one right and one left dP_4_ (IPS-82796; IPS-82797); one left P_4_ (IPS-82806); two complete and one broken left M_1_ (IPS-82800; IPS-82801; IPS-82802); one complete left M_2_ and one fragment of a right M_2_ (IPS-82805; IPS-82798); two left M_3_ (IPS-82803, IPS-82804); one right dP^4^ (IPS-82814); one entire and one broken left M^1^ (IPS-82808; IPS-82809); one left M^2^ (IPS-82815); and one left M^3^ (IPS-82799), all from Masia de l’Hereuet.

*Occurrence.* Masia de l’Hereuet, Àger sub-basin (Southern Pyrenean Basins, Lleida province, NE Spain); early Eocene (Neustrian, MP8+9, Mammal Paleogene Reference Level).

**Figure 3:**
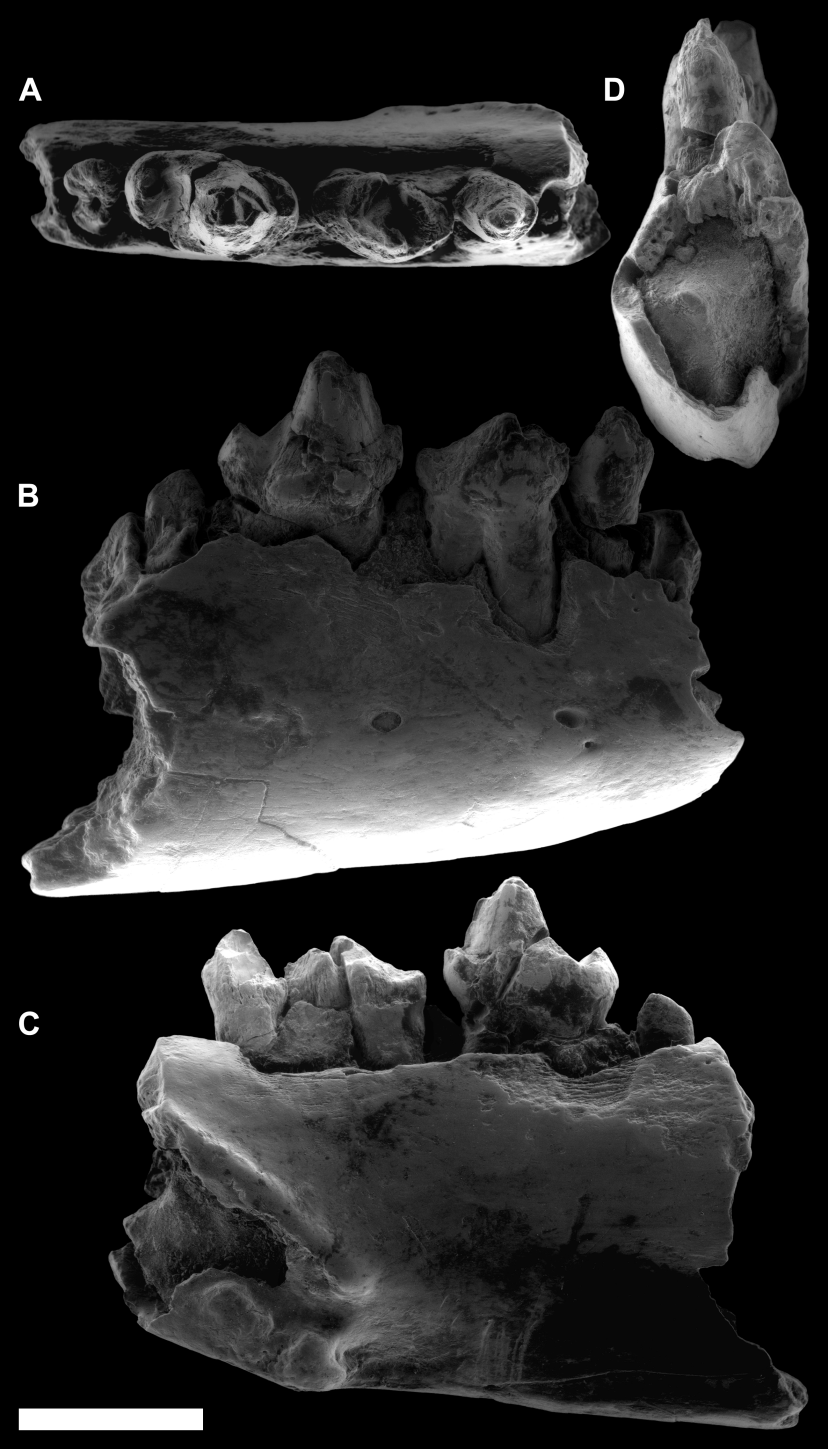
ESEM images of *Agerinia marandati* sp. nov. from Masia de l’Hereuet. Right mandible (IPS-82807) with alveoli of the canine and P_1_, premolars from P_2_ to P_4_, and mesial root of the M_1_ in occlusal (A), buccal (B), lingual (C) and mesial (D) views. Scale bar represents 3 mm.

**Figure 4:**
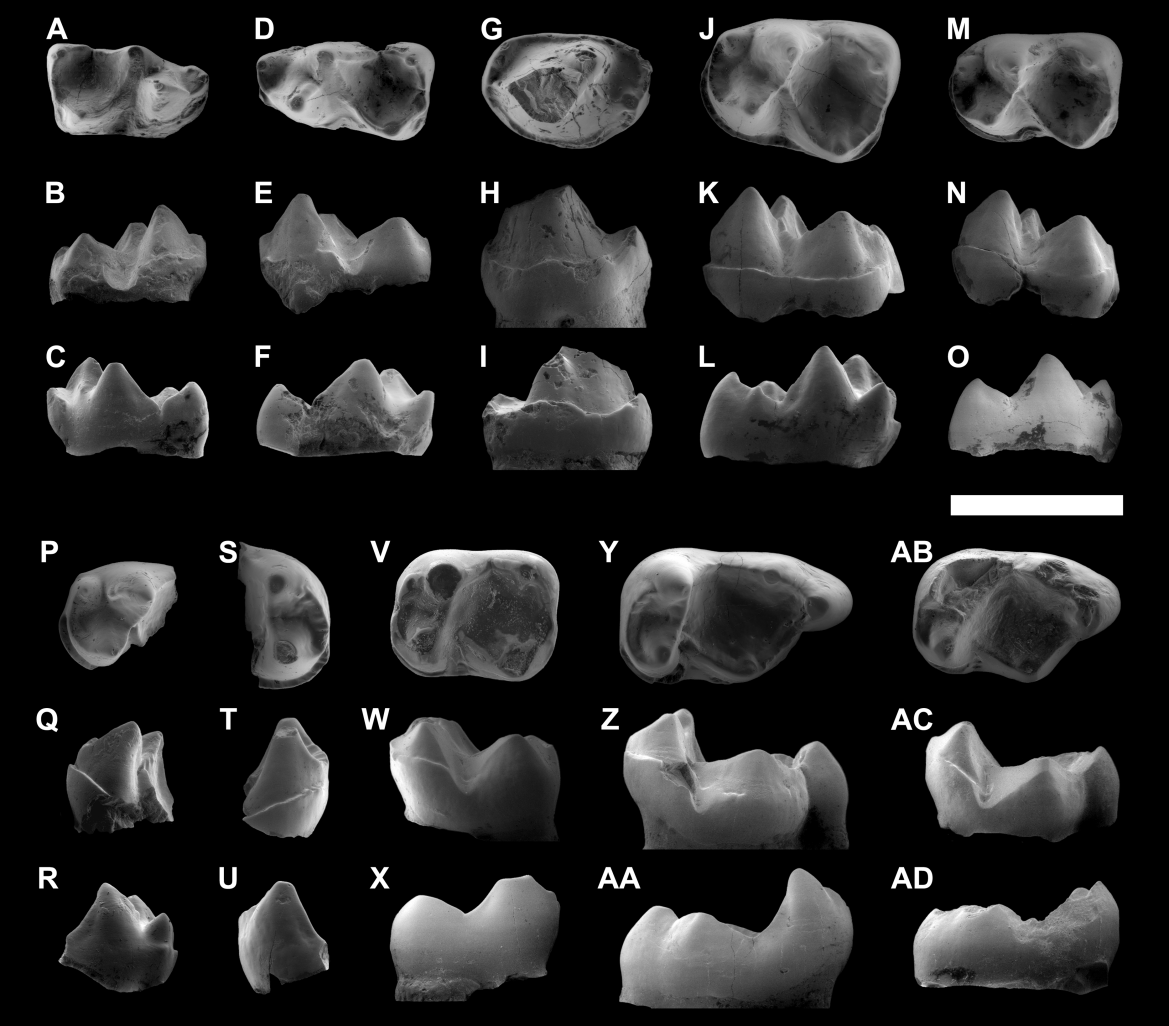
ESEM images of isolated lower teeth of *Agerinia marandati* sp. nov. from Masia de l’Hereuet. IPS-82796, right dP_4_ in occlusal (A), buccal (B) and lingual (C) views. IPS-82797, left dP_4_ in occlusal (D), buccal (E) and lingual (F) views. IPS-82806, left P_4_ in occlusal (G), buccal (H) and lingual (I) views. IPS-82801 (holotype), left M_1_ in occlusal (J), buccal (K) and lingual (L) views. IPS-82800, left M_1_ in occlusal (M), buccal (N) and lingual (O) views. IPS-82802, fragment of left M_1_ in occlusal (P), buccal (Q) and lingual (R) views. IPS-82798, fragment of right M_2_ in occlusal (S), buccal (T) and lingual (U) views. IPS-82805, left M_2_ in occlusal (V), buccal (W) and lingual (X) views. IPS-82803, left M_3_ in occlusal (Y), buccal (Z) and lingual (AA) views. IPS-82804, left M_3_ in occlusal (AB), buccal (AC) and lingual (AD) views. Scale bar represents 3 mm.

**Figure 5:**
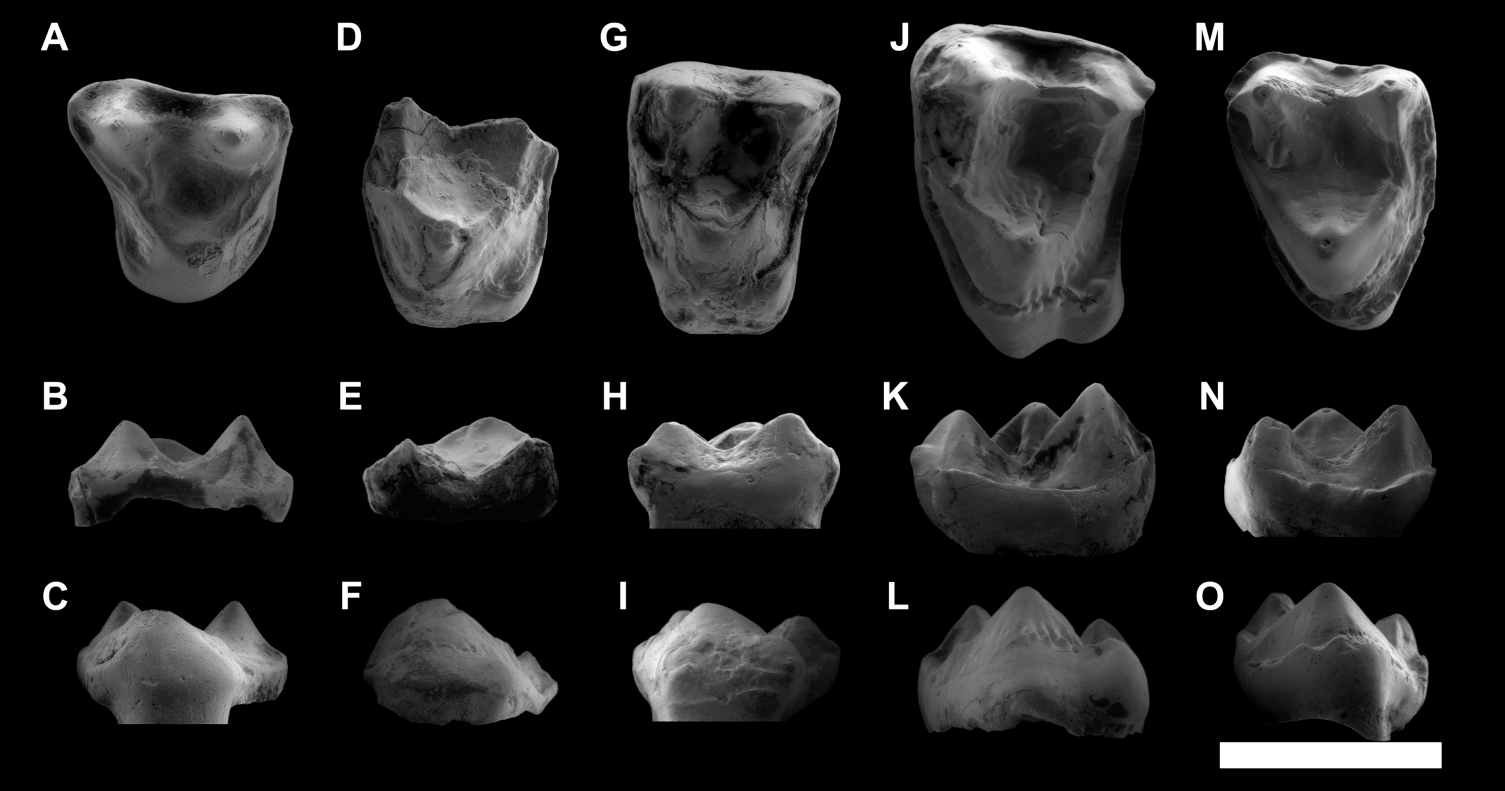
ESEM images of isolated upper teeth of *Agerinia marandati* sp. nov. from Masia de l’Hereuet. IPS-82814, right dP^4^ in occlusal (A), buccal (B) and lingual (C) views. IPS-82809, fragment of left M^1^ in occlusal (D), buccal (E) and lingual (F) views. IPS-82808 left M^1^ in occlusal (G), buccal (H) and lingual (I) views. IPS-82815, left M^2^ in occlusal (J), buccal (K) and lingual (**L**) views. IPS-82799, left M^3^ in occlusal (M), buccal (N) and lingual (O) views. Scale bar represents 3 mm.

*Diagnosis.* Medium-sized notharctid. P_1_ and P_2_ single-rooted. P_4_ with well-developed protoconid and metaconid, distinct paraconid, hypoconid and cristid obliqua. M_1_ with a voluminous paraconid, open trigonid basin, trigonid clearly narrower than the talonid, protocristid oblique to the lingual and buccal borders. M_2_ and M_3_ without paraconid, closed trigonid basin, trigonid nearly equal in width to the talonid, and a protocristid subperpendicular to the lingual and buccal borders. Upper molars with paraconule more developed than the metaconule, and without pericone. M^1^ and M^2^ with distinct hypocone and well-developed preparaconule crista and hypoparacrista, joining paracone and paraconule.

*Differential diagnosis*. *Agerinia marandati* differs from *A. roselli* in having a less molarized P_4_ (lacking an entoconid), a relatively large M_1_ paraconid, an open M_1_ trigonid basin and a protocristid that is more oblique to the lingual and buccal borders on the M_1_. *Agerinia marandati* differs from *A. smithorum* in having a single-rooted P_2_, a more molarized P_4_ (showing distinct paraconid and hypoconid) and in lacking a paraconid on the M_2_. *Agerinia marandati* differs from *Pronycticebus gaudryi* in the less bulbous cuspids, the single-rooted P_1_, the absence of paraconid on the M_2_ and M_3_, and the less developed hypocone, parastyle and metastyle on the upper molars. It further differs from *Europolemur* in the smaller size and in the much more developed paraconid on the M_1_. It differs from *Protoadapis* in the much less robust cusps, in having P_3_ and P_4_ similar in height and in the well-developed paraconid on the M_1_. *Agerinia marandati* differs from *Cantius eppsi* and *Cantius savagei* in its smaller size and less inflated cusps. It further differs from *Cantius eppsi* in the lack of paraconid on the M_2_ and M_3_, in the better-developed hypocone, hypoparacrista and hypometacrista and the less-developed paraconule, metaconule and lingual cingulum on the upper teeth. Furthermore, it differs from *Cantius savagei* in the narrower M_1_ and in the slightly longer talonid basin. Besides, *Agerinia marandati* differs from *Marcgodinotius indicus* in its smaller size, in the single-rooted P_2_, the differentiated paraconid and metaconid on the P_4_, the lack of paraconid on the M_2_ and M_3_, and in the less concave mesial and distal borders in the upper molars. *A. marandati* differs from *Asiadapis cambayensis* in the presence of a P_1_, the better-developed paraconid on the M_1_, and in the more developed hypocone, paraconule and metaconule on the upper molars.

*Measurements.* See Table 1.

**Table 1:** Teeth measurements (in mm) of *Agerinia marandati* sp. nov. from Masia de l’Hereuet.

Catalogue number	Tooth	Length	Width
IPS-82796	dP_4_	2.98	1.61
IPS-82797	dP_4_	3.18	1.81
IPS-82807	P_2_	≥1.58	≥1.22
	P_3_	≥2.68	≥1.61
	P_4_	≥3.16	≥1.99
IPS-82806	P_4_	3.21	2.25
IPS-82800	M_1_	3.11	2.14
IPS-82801	M_1_	3.70	2.60
IPS-82802	M_1_	–	–
IPS-82805	M_2_	3.05	2.50
IPS-82798	M_2_	–	–
IPS-82803	M_3_	4.18	2.51
IPS-82804	M_3_	3.55	2.36
IPS-82814	dP^4^	3.13	3.19
IPS-82808	M^1^	3.00	3.86
IPS-82809	M^1^	–	–
IPS-82815	M^2^	3.72	4.68
IPS-82799	M^3^	2.94	3.83

### Description

*Mandible.* This specimen preserves the distal part of the canine alveolus, which is placed just mesially with respect to the P_1_, with no diastema between these two teeth. The P_1_ root is preserved, which corresponds to a small premolar, and is aligned with the rest of the teeth on the longitudinal axis of the mandible. The mandible preserves all the teeth from P_2_ to P_4_ and the mesial root of the M_1_; however, they are all damaged. Furthermore, on the lingual side of the mandible, below the P_1_ and P_2_, a slightly protruding stripe can be observed descending from the mesial to the distal part, probably indicating an unfused mandible. On the buccal side of the mandible there are three mental foramina of similar size and a tiny one mesially situated just on the bottom of the central one. The mesial-most foramen is broken and placed between the alveoli of the canine and P_1_. The central foramina are situated between the root of the P_2_ and the mesial root of the P_3_ and the distal-most foramen is placed underneath the mesial root of the P_4_.

*dP*_4_. The trigonid is elongated, much longer than wide; it is slightly shorter and clearly narrower than the talonid. There is a broad space separating the paraconid and metaconid. The trigonid is open lingually. The paraconid is well differentiated, clearly smaller than protoconid and metaconid, and placed in the mesiolingual extreme of the tooth. The paracristid runs buccally from the paraconid and curves distally reaching the protoconid. The protoconid is located mesially with respect to the metaconid. Both cuspids are similar in height and connected by a protocristid, oblique to the buccal and lingual borders. There is no premetacristid. The cristid obliqua reaches the apex of the metaconid. The talonid basin is deep. The hypoconid is placed mesially with respect to the entoconid. The postcristid is thickened at its central part, but it does not bear a distinct hypoconulid. There is a well-marked notch that separates the preentocristid and the postmetacristid. The buccal cingulid is slightly marked and restricted to the mesiobuccal corner of the tooth, from the buccal base of paraconid to the distal base of protoconid. There are two roots.

*P*_2_. The P_2_ is slightly longer than broad, with an oval outline. It is larger than P_1_ (based on the size of the roots), and clearly smaller than P_3_ and P_4_. The morphology is rather simple, with a single distinguishable cuspid (protoconid) and two sharp cristids directed mesially and distally from the apex to the base of the crown. At the distal end of the tooth, there is a small but distinguishable bulge centrally located. There is a weak cingulid on the mesial part of the tooth, and a more developed cingulid occupying the distolingual and the distobuccal borders. There is only one root.

*P*_3_. There is only one P_3_ known, twice long as it is broad. The crown is strongly damaged, preventing the observation of the dental morphology. However some traits can be described, particularly the presence of a protuberance on the distal end of the crown, centrally located, that does not constitute a clear cuspid. The buccal and lingual cingulids are faintly marked and restricted to the mesial and distal ends of the tooth. On the distolingual corner, the lingual cingulid encloses an incipient and shallow talonid basin. There are two roots.

*P*_4_. The P_4_ is larger than P_3_. The outline is oval, somewhat more mesiodistally elongated in specimen IPS-82807 than in IPS-82806. There is a distinct paraconid in mesiolingual position. The paracristid descends from the protoconid apex, curving lingually at its end and reaching the paraconid. The metaconid, clearly distinguishable, is attached to the distolingual side of the protoconid in specimen IPS-82806 (the poor preservation of specimen IPS-82807 prevents the observation of this trait). There is a distinct cristid obliqua that runs from the joint between protoconid and metaconid to the hypoconid, which is centrally located at the distal side of the tooth (this trait is also only observed in specimen IPS-82806). The hypoconid is as high as the paraconid. The buccal and lingual cingulids, high and well marked, surround the entire base of the tooth in specimen IPS-82806; in IPS-82807 these cingulids seem less marked, but it can be due to the bad preservation of this specimen. The lingual cingulid encloses a shallow talonid basin, restricted to the distolingual corner. There are two roots.

*M*_1_. The trigonid is shorter and much narrower than the talonid. The trigonid basin is as long as it is wide and it is open between the paraconid and the metaconid. The paraconid is well differentiated but clearly smaller than the other trigonid cuspids; it is placed on the mesiolingual corner of the tooth, closer to the metaconid than to the protoconid. The paracristid runs mesially from the protoconid and, at the mesiobuccal corner of the tooth, curves buccally reaching the paraconid. The protoconid is located in a more mesial position than the metaconid. Both cuspids are connected by a protocristid, clearly oblique to the lingual and buccal borders, that shows a well-marked V-shaped valley. In the specimens IPS-82800 and IPS-82801, there is no premetacristid, whereas IPS-82802 shows a cristid directed mesially from the metaconid, which does not reach the paraconid. In this latter specimen, the metaconid apex is slightly curved distally. The cristid obliqua reaches the trigonid wall at the level of the buccal base of the metaconid. The talonid basin is clearly deeper than the trigonid basin. The hypoconid is placed mesially with respect to the entoconid. The postcristid connects the hypoconid and entoconid, which shows several enamel swellings that do not constitute a distinct hypoconulid. The preentocristid connects to the postmetacristid closing the talonid lingually. IPS-82801 shows a small but well differentiated bulge on the middle of the preentocristid. The buccal cingulid runs from the mesiobuccal base of the paraconid to the distolingual base of the hypoconid. This cingulid is very strong on the mesial border and below the protoconid, and weaker at the level of the talonid, being interrupted below the hypoconid in IPS-82800.

*M*_2_. The trigonid is wider than long; it is much shorter than the talonid, but similar in width, and therefore the outline of the tooth is rectangular. There is no paraconid. The paracristid runs from the protoconid, surrounding the mesial border and continuing into a premetacristid that reaches the metaconid, closing the trigonid basin lingually. The protoconid is located in a slightly more mesial position than the metaconid, so the protocristid is almost perpendicular to the buccal and lingual borders of the tooth. The cristid obliqua reaches the trigonid wall close to the lingual base of protoconid. The talonid basin is closed, moderately shallow and as wide as it is long. The hypoconid is slightly more voluminous than the entoconid and placed in a slightly more mesial position. The postcristid joins hypoconid and entoconid; at its distobuccal part, it thickens forming a tiny hypoconulid. The preentocristid and postmetacristid are connected, closing the talonid basin lingually. The buccal cingulid is weak and restricted to the base of the protoconid.

*M*_3_. The trigonid is much shorter than the talonid and similar in width. There is no paraconid. The paracristid continues into a premetacristid reaching the metaconid, so the trigonid basin is closed. The protoconid is placed in a slightly more mesial position than the metaconid. The protocristid is nearly perpendicular to the buccal and lingual margins of the tooth. The cristid obliqua is straight and reaches the trigonid wall in a more buccal position than in the M_2_. The talonid basin is closed, moderately deep and longer than wide. The hypoconid is slightly more voluminous and placed more mesially than the entoconid. Preentocristid and postmetacristid are connected, closing the talonid buccally. The hypoconulid lobe is prominent, but longer and better differentiated from the talonid basin in IPS-82803 than in IPS-82804. The postentocristid shows a slightly marked valley between the hypoconulid and the entoconid in specimen IPS-82803. The buccal cingulid is visible on the base of the protoconid. On IPS-82803 a very weak cingulid is hardly observed on the buccal base of the hypoconid.

*dP*^4^. The specimen is eroded, lacking the enamel. The outline is subtriangular, with the buccal side longer than the lingual side and it has three main cusps: paracone, metacone and protocone. There is no hypocone. The trigon basin is quite deep. The preparacrista is slightly marked and runs from the apex of the paracone, curving buccally, reaching the mesiobuccal corner, which is broken. The postparacrista runs distally from the paracone and connects to the premetacrista, which reaches the metacone. The postmetacrista is straight and connects the metacone to the distobuccal corner of the tooth. The protocone is slightly lower than paracone and metacone. The preprotocrista connects the protocone to a small paraconule. The preparaconule crista runs around the mesiobuccal half of the tooth, from the paraconule to the mesiobuccal corner. The metaconule is more developed than the paraconule. Three cristae run from the metaconule: a well-marked postprotocrista that connects to the protocone, a short premetaconule crista, which is directed to the metacone but does not reach its apex, and a longer postmetaconule crista (also known as lateral posterior transverse crista) that borders the distal margin of the tooth and reaches the postmetacrista at the distobuccal corner of the tooth. There is a faint anterocingulum that runs from the mesiolingual base of the protocone to the mesial half of the tooth, reaching the preparaconule crista. The postcingulum is restricted to the distolingual corner of the tooth, reaching the postmetaconule crista. There are three roots.

*M*^1^. The outline is subtriangular, being much wider than long and with the buccal side somewhat longer than the lingual one. The trigon basin is deep. The paracone is somewhat larger than the metacone; the protocone is slightly lower than the buccal cusps, and the hypocone is clearly smaller and lower than the rest. The preparacrista is weak. From the apex of the paracone, a straight postparacrista descends distally and connects with the premetacrista that reaches the metacone apex. The postmetacrista is straight and runs from the apex of metacone to the distobuccal corner of the tooth. The paraconule is much more developed than the metaconule; it has two buccal cristae: a preparaconule crista, reaching the end of the preparacrista at the mesiobuccal corner of the tooth, and a hypoparacrista that reaches the apex of the paracone. These cristae enclose a well-developed basin mesiolingual to the paracone. The preprotocrista is well marked and connects the paraconule and the protocone. The metaconule is connected to the metacone by a hypometacrista and to the protocone by a postprotocrista. There is a small but distinct hypocone at the distolingual corner of the tooth; it is weakly connected to the base of the protocone by a very short and low postprotocingulum. There is a weak buccal cingulum restricted to the central part of the buccal border, between paracone and metacone. The anterocingulum is marked and connects to the preparaconule crista; it is longer in IPS-82809 than IPS-82808, surrounding the mesiolingual corner in the former. The posthypocone crista continues in a long and well-marked postcingulum that occupies the entire distal border, being interrupted at the distobuccal corner, without connecting to the postmetacrista. The specimen IPS-82808, shows a slightly wrinkled enamel surface at the lingual base of the protocone.

*M*^2^. It is larger than the M^1^, subtriangular, with the lingual side slightly shorter than the buccal side. The trigon basin is very deep. The paracone is slightly larger than the metacone and similar in height to the protocone. The preparacrista is well marked and straight; it connects the paracone to a small parastyle. The postparacrista and premetacrista are sharp and descend strongly, so their connection is placed in a very low position. The postmetacrista descends from the apex of the metacone and curves slightly towards the buccal side, reaching the distobuccal corner of the tooth. It thickens at its distal end, forming an incipient metastyle. Paraconule and metaconule are similar in size. From the paraconule, a preparaconule crista borders the mesial part of the tooth, reaching the parastyle. There is a deep basin mesiolingual with respect to the paracone, enclosed by the preparaconule and hypoparacrista. From the protocone, the preprotocrista and the postprotocrista connect to the paraconule and the metaconule, respectively. The hypometacrista is well marked and joins the metaconule with the metacone. There is a well-developed hypocone, placed in a marginal position at the distolingual corner of the tooth and protruding strongly on the outline of the molar. It is connected to the distal base of protocone by a short postprotocingulum. There is no pericone. The buccal cingulum is well developed and runs from the parastyle to the distobuccal corner of the tooth, without meeting the metastyle. The anterocingulum is also strong; it starts at the mesiolingual corner of the tooth and connects to the preparaconule crista. The anterocingulum continues into a lingual cingulum, which is crenulated at the base of the protocone. The posthypocone crista continues in a postcingulum that extends to the distobuccal corner of the tooth, without connecting to the metastyle. This cingulum encloses a very small talon basin. This specimen shows slight enamel wrinkling, especially on the trigon basin and on the distolingual base of protocone.

*M*^3^. The outline is triangular. The paracone is notably larger than the metacone and similar in height to the protocone. The trigon basin is deep. There is no hypocone. The preparacrista is curved lingually and connects the paracone to a hardly distinct parastyle. The postparacrista and premetacrista are well marked. The postmetacrista is very weak and curved buccally; it reaches the distobuccal corner of the tooth, without forming a metastyle. The conules are much less developed than in the M^1^ and M^2^: the paraconule is a mere thickening of the preprotocrista, and the metaconule is absent. The preparaconule crista is sharp and long, reaching the parastyle. The hypoparacrista is weaker than the preparaconule crista; it descends lingually from the paracone but does not reach the paraconule. The postprotocrista connects the protocone and the metacone. The buccal cingulum starts at the mesiobuccal base of the paracone, without reaching the parastyle. This cingulum is strong at the base of paracone, but weak and discontinuous at the base of metacone. The anterocingulum starts at the base of the paraconule, surrounds the mesiolingual border of the tooth and continues in a strong postcingulum that occupies the whole distal border. This cingulum shows some bulges at its lingual base that do not constitute distinct cuspids. The enamel is slightly wrinkled on the distolingual side.

**Figure 6:**
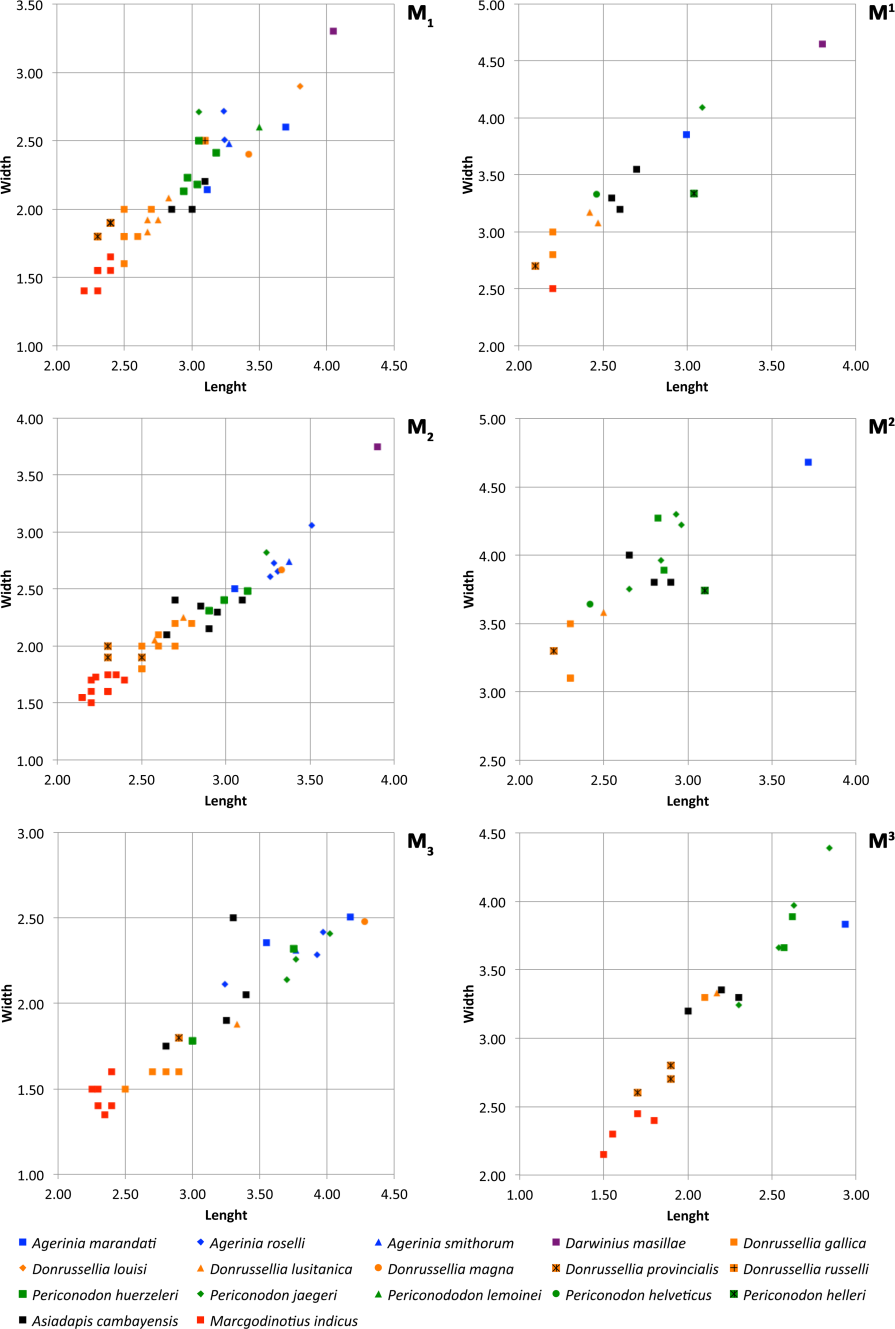
Size graphs (length × width, in mm) of the molars of the different species of *Agerinia*, *Darwinius*, *Donrussellia*, *Periconodon*, *Asiadapis* and *Marcgodinotius.* Measurements of *Agerinia marandati* are those presented in this work. Measurements of *Periconodon helveticus* have been taken directly on high-resolution casts. Data of *Agerinia roselli* and *Agerinia marandati* after Femenias-Gual et al. (2016a) and Femenias-Gual et al. (2016b), respectively; data of *Darwinius masillae* after Franzen et al. (2009); data of *Donrussellia gallica* after Russell, Louis & Savage (1967); data of *Donrussellia louisi*, *Donrussellia russelli, Periconodon lemoinei* and *Periconodon huerzeleri* after Gingerich (1977); data of *Donrussellia lusitanica* after Estravís (2000); data of *Donrussellia magna* after Godinot et al. (1987); data of *Donrussellia provincialis* after Godinot (1981); data of *Periconodon jaegeri* after Godinot (1988); data of *Periconodon helleri* after Thalmann (1994); data of *Asiadapis cambayensis* and *Marcgodinotius indicus* after Rose et al. (2007) and Rose et al. (2009).

### Comparisons

*Comparisons with other samples attributed to Agerinia*. The new species *Agerinia marandati* is quite similar in size and in some morphological traits to *Agerinia roselli* from Les Saleres (Fig. 6; Crusafont-Pairó, 1967; Szalay, 1971; Femenias-Gual et al., 2016a) such as the single-rooted P_2_, the distinct paraconid on the P_4_ or the lack of paraconid on the M_2_. However, *A. marandati* clearly differs in other several traits. The root of the P_1_ of *A. marandati* is centrally located on the mesiodistal axis of the mandible, while in *A. roselli* it is clearly shifted buccally (here, we consider that the most mesial root of *A. roselli* described by Femenias-Gual et al. (2016a) corresponds to the P_1_, see Discussion). The metaconid and hypoconid of the P_4_ are slightly better differentiated in *A. roselli* than in *A. marandati*. In addition, the P_4_ of *A. marandati* lacks the distinct entoconid that is present in *A. roselli*. Besides, the M_1_ paraconid is clearly larger in *A. marandati*, while it is very small in *A. roselli*. Finally, the distal and intermediate mental foramina of *A. roselli* are clearly more mesially located than in *A. marandati.*

Regarding *Agerinia smithorum* from Casa Retjo-1 (Femenias-Gual et al., 2016b), it is also very similar in size to *A. marandati* (Fig. 6). Furthermore, both species share several traits such as the central position of the P_1_ on the mesiodistal axis of the mandible, the lack of entoconid on the P_4_, the well-developed paraconid on the M_1_, or the similar disposition of the distal and intermediate mental foramina, this latter being only a little more mesially located in *A. marandati* than in *A. smithorum.* Nevertheless, there are several differences between these species. The P_1_ alveolus is more compressed mesiodistally in *A. marandati* than in *A. smithorum*, thus suggesting a more reduced premolar (the P_1_ crown is not preserved in these species). The number of roots of P_2_ is different, being double-rooted in *A. smithorum* and single-rooted in *A. marandati*. Additionally, the latter species differs from *A. smithorum* in the molarization of the P_4_. *A. marandati* shows a well developed metaconid and distinct paraconid and hypoconid, while in *A. smithorum* the metaconid is smaller and the paraconid and hypoconid are absent*.* Furthermore, *A. marandati* lacks the paraconid on the M_2_, while *A. smithorum* shows a tiny one. Finally, the mesial-most mental foramen is clearly more mesially located in *A. marandati* than in *A. smithorum.*

Godinot (1983) described two partial mandibles of *Agerinia* cf. *roselli* from Azillanet (MP10, France), which are similar in size or slightly larger than *A. marandati.* Furthermore, the M_1_ from Azillanet clearly differs from that of *A. marandati* in lacking the paraconid.

Peláez-Campomanes (1995) documented the presence of *Agerinia* sp. in the fossil site Casa Ramón (MP11, N Spain), which is clearly smaller than *A. marandati.* Moreover, they differ in several traits. In the M_1_ from Casa Ramón, the paracristid forms an acute angle in the mesiobuccal corner; this angle is obtuse in *A. marandati.* The M_2_ of *Agerinia* sp. is proportionally narrower than that of *A. marandati,* and the paracristid of the latter is higher than in the specimen from Casa Ramón. Besides, the protocristid of *A. marandati* is more perpendicular to the buccal and lingual borders of the tooth than in *Agerinia* sp. from Casa Ramón. The M^1−2^ of Casa Ramón lacks the hypoparacrista that is well marked in the M^1^ and M^2^ of *A. marandati*. Finally, the hypocone is connected to the distal base of protocone by a short postprotocingulum in the M^1^ and M^2^ of *A. marandati* but it is isolated in *Agerinia* sp.

Herbomel & Godinot (2011) described some specimens from Condé-en-Brie (MP8+9, France), preliminarily assigned to *Agerinia* sp. This form is, in general terms, somewhat larger than *A. marandati* and shows slightly more bulbous cusps. The paraconid of the M_1_ is larger in *A. marandati* than in *Agerinia* sp. from Condé-en-Brie, where it varies from small to moderate. The M^1^ and M^2^ of *A. marandati* show a hypoparacrista that connects the paracone with the paraconule, while in *Agerinia* sp. there is only a short hypoparacrista that does not reach the paraconule. Furthermore, the hypocone of *Agerinia* sp. varies in size from large to small or even absent in many M^1^ and M^2^, whereas it is well marked in the M^1^ and M^2^ of *A. marandati*. The pericone is absent in the upper molars of *A. marandati*, while in some M^2^ from Condé-en-Brie the anterior cingulum thickens forming a real pericone. The M^3^ of *Agerinia* sp. frequently display one or two lingual cusps; on the contrary, in the M^3^ of *A. marandati* there are some bulges at the level of the connection of the anterocingulum and the postcingulum, which do not constitute distinct cusps.

There is a single M_2_ from Rians (MP7, France) described by Godinot (1983), which was determined as cf. *Agerinia.* This specimen is clearly larger than the M_2_ of *A. marandati* and shows some morphological differences. For instance, the difference in width between the trigonid and the talonid is much more marked in the M_2_ of cf. *Agerinia* than in that of *A. marandati,* which shows a more squared outline. Furthermore, cf. *Agerinia* shows a well-developed paraconid that is absent in the M_2_ of *A. marandati.* Finally, the M_2_ from Rians shows an expansion of the distolingual corner and has the entoconid more distally placed with respect to the hypoconid than in *A. marandati*.

*Comparisons with other Eurasian Notharctidae*. The new species *A. marandati* has been compared to *Periconodon* and *Darwinius*, which together with *Agerinia,* form a close taxonomic group following Godinot (2015). Moreover, it has been compared with *Donrussellia*, the only other Euprimate genus found in the Iberian Peninsula in the early Eocene, as well as with other Eocene Eurasian notharctids, such as the genera *Pronycticebus, Europolemur, Protoadapis, Cantius, Marcgodinotius* and *Asiadapis*.

Although the taxonomy of *Periconodon* is uncertain, five species and a specimen without specific attribution constitute this genus (following Godinot, 1988; Godinot, 2015): *P. helveticus* from Ergerkingen (MP13; Rütimeyer, 1891), *P. lemoinei* from Grauves (MP10, Gingerich, 1977), *P. huerzeleri* from Bouxwiller (MP13, Gingerich, 1977), *P. helleri* from Geiseltal (MP13-14, Schwartz, Tattersall & Haubold, 1983), *P. jaegeri* from Bouxwiller (MP13, Godinot, 1988) and *Periconodon* sp. from Eckfeld Maar (MP13, Franzen, 2004). Regarding size, the teeth of *Agerinia marandati* are larger than those of *P. huerzeleri* and *P. helveticus*, similar in size to those of *P. jaegeri* and *P. lemoinei,* and shorter and broader than those of *Periconodon* sp. from Eckfeld Maar (Fig. 6; there are no available published measurements of *P. helleri*). In any case, *A. marandati* shows strong morphological differences with the genus *Periconodon,* including the lack of pericone on the upper molars, the better differentiated metaconid on the P_4_, the presence of a distinct paraconid on the M_1_ and of an entoconid on the M_3_ (Franzen, 2004; Godinot, 2015).

Regarding *Darwinius masillae* from Messel (MP11, Franzen et al., 2009), it is clearly larger than *A. marandati* (Fig. 6) and lacks the first lower premolar. The upper molars of *D. masillae* lack the metaconule that is present in *A. marandati*; the paraconule is barely marked in *Darwinius*. The hypoparacrista is faint and does not reach the paraconule. Moreover, the talon basin is clearly broader in the upper molars of *Darwinius* than in those of *A. marandati*. The anterocingulum and postcingulum of *D. masillae* are more developed than those of *A. marandati.* The M_1_ of *Darwinius* lacks a paraconid and shows a closed trigonid basin, whereas in *A. marandati* this tooth shows an open trigonid basin with a well-differentiated paraconid. Furthermore, the M_1_ of *Darwinius* shows a well-differentiated metastylid that is absent in *A. marandati.* Finally, the mandible of *D. masillae* shows only one mental foramen below the P_2_, whereas there are three foramina on *A. marandati.*

Furthermore, *A. marandati* clearly differs from the genus *Donrussellia*, which includes six species: *D. gallica, D. russelli* and *D. louisi* from Avenay, France (MP8+9, Russell, Louis & Savage, 1967; Gingerich, 1977), *D. provincialis* from Rians, France (MP7, Godinot, 1978), *D. magna* from Palette, France (MP7, Godinot et al., 1987) and *D. lusitanica* from Silveirinha, Portugal (MP7, Estravís, 2000). Concerning the size, *A. marandati* is clearly larger than *Donrussellia provincialis, D. gallica* and *D. lusitanica* and similar in size or slightly smaller than *D. magna, D. russelli* and *D. louisi* (Fig. 6)*.* Regarding the morphology, *Donrussellia* clearly differs from *A. marandati* in the presence of a well-developed paraconid in all lower molars, whereas *A. marandati* only shows a well-marked cuspid in the M_1_. Furthermore, *Donrussellia* differs from *A. marandati* in having a double-rooted P_2_ while in the latter it is single rooted. In the M_1_ of *Donrussellia* the trigonid is as long as the talonid, while in *A. marandati* it is shorter than the talonid. The M^1^ and M^2^ of *A. marandati* clearly differ from those of *Donrussellia* in having a more developed hypoparacrista*.* Besides, the hypocone is distinct in the M^1^ and M^2^ of *A. marandati,* whereas this cusp is only present in some M^2^ of *Donrussellia*.

Additionally, *A. marandati* is smaller than *Pronycticebus gaudryi* from Mermerlein in France (MP20, Grandidier, 1904; Szalay, 1971). The cusps are generally more bulbous in *P. gaudryi* than in *A. marandati.* Regarding the lower teeth, they differ in the number of roots of the P_2_, being double-rooted in *P. gaudryi* and single-rooted in *A. marandati*. Besides, the former species shows a paraconid in all lower molars, whereas *A. marandati* only displays a paraconid in the M_1_. The upper teeth of *A. marandati* show less developed hypocone, parastyle and metastyle than those of *P. gaudryi.* Furthermore, the trigon basin of *A. marandati* is wider than in *P. gaudryi*, in which it is as long as it is wide. Moreover, the M^3^ of *P. gaudryi* displays a hypocone, and the hypoparacrista reaches the paraconule, whereas in the M^3^ of *A. marandati* the hypoparacrista does not join the paraconule and the hypocone is absent.

The genus *Europolemur* includes four species following Godinot (2015): *E. koenigswaldi* and *E. kelleri* from Messel (MP11, Franzen, 1987; Franzen, 2000), *E. klatti* from Geiseltal (MP13, Thalmann, 1994) and *E. dunaifi* from Bouxwiller (MP13, Tattersall & Schwartz, 1983; Godinot, 1988). All these species are much larger than *A. marandati.* The paraconid is much more developed in the M_1_ of *A. marandati* than in *Europolemur*, in which this cuspid can be small or absent. Furthermore, among other differences, *E. koenigswaldi* and *E. klatti* lack the P_1_ whereas *A. marandati* preserves it. Besides, *E. klatti* shows a double-rooted P_2_, while this premolar is single rooted in *A. marandati.* The size of the hypocone is variable in the upper molars of *Europolemur,* being less developed in *E. kelleri* than in *A. marandati,* and more developed in *E. dunaifi* than in *A. marandati.*

The genus *Protoadapis* comprises six species according to Godinot (2015): *Protoadapis angustidens* and *Protoadapis brachyrhynchus* from unknown levels of the Quercy phosphorites (Russell, Louis & Savage, 1967; Gingerich, 1975), *Protoadapis curvicuspidens* from different sites including Grauves (MP10, Russell, Louis & Savage, 1967) and *Protoadapis weigelti, Protoadapis ignoratus* and *Protoadapis muechelnensis* from Geiseltal (MP12, Thalmann, 1994). All these species are poorly known and only represented by lower teeth, except *P. curvicuspidens*. In any case, there are several important differences between *Protoadapis* and *A. marandati*. The genus *Protoadapis* is much larger and displays more robust cusps than *Agerinia*. In addition, the P_3_ is clearly higher than the P_4_ in *Protoadapis*, whereas in *Agerinia* these premolars are more similar in height. Moreover, the paraconid of the lower molars of *Protoadapis* is shown as a residual cuspule, whereas *A. marandati* displays a well-developed paraconid on the M_1_.

Several species are included in the genus *Cantius*, but only two are recorded from Europe: *Cantius eppsi* from Abbey Wood (MP8+9; Hooker, 2010) and *Cantius savagei* from Muntigny and Avenay (MP8+9; Gingerich, 1977). These two species show notable differences with *A. marandati.* Both *C. eppsi* and *C. savagei* are clearly larger and show more inflated cusps than *Agerinia.* Besides, *Agerinia marandati* differs from *C. eppsi* in having a paraconid only on the M_1_, whereas the latter generally shows a well-developed paraconid in all lower molars. Regarding the upper molars, *A. marandati* shows better-developed hypocone, hypoparacrista and hypometacrista than *C. eppsi*. In addition, *A. marandati* has slightly wrinkled enamel on the M^2^ an M^3^, whereas the teeth of *C. eppsi* have smooth enamel. Besides, *C. eppsi* shows a postprotocingulum, while in *A. marandati* it is absent. Furthermore, *C. eppsi* displays more developed paraconule, metaconule and lingual cingulum than *A. marandati.* The M_1_ of *Cantius savagei* is broader than that of *A. marandati* and displays a slightly shorter talonid basin.

The asiadapine *Marcgodinotius indicus* from Vastan and Tadkehwar mines (early Eocene, India; Bajpai et al., 2007; Rose et al., 2007; Rose et al., 2009; Smith et al., 2016) differs from *A. marandati* in several traits. Regarding the size, *A. marandati* is larger than *M. indicus* (Fig. 6). *Marcgodinotius* has a double-rooted P_2_, whereas in *A. marandati* this premolar is single rooted. Moreover, the P_4_ of *A. marandati* displays small but differentiated paraconid and metaconid, whereas the P_4_ of *M. indicus* usually lacks these cuspids. Some specimens of *M. indicus* show a low metaconid posterolingually placed in relation to the protoconid; on the contrary, in the P_4_ of *A. marandati* the metaconid is higher than in *Marcgodinotius* and lingually attached to the protoconid. The lower molars of *M. indicus* display a slighly longer trigonid than those of *A. marandati.* Furthermore, the M_1_ and M_2_ of *M. indicus* show a small, low and buccally shifted paraconid, while *A. marandati* only displays a well-developed paraconid on the M_1_. Furthermore, the outline of the talonid basin of *A. marandati* is clearly more rounded than in *M. indicus*. Regarding the upper teeth, the decidual P^4^ of *A. marandati* lacks the hypocone and the hypoparacrista that are well marked in *M. indicus.* The difference in length between the buccal and the lingual sides is less marked in the upper molars of *A. marandati* than in *M. indicus*. The outline of the upper molars is also different, showing concave mesial and distal borders in *M. indicus*. Besides, *M. indicus* differs from *A. marandati* in having a more marked buccal cingulum and styles in the M^1^. The M^3^ of *M. indicus* is much wider than that of *A. marandati* and also differs from the latter in the more developed parastyle and in the presence in some specimens of a small and low paraconule, premetaconule and postmetaconule cristae.

Concerning *Asiadapis cambayensis* from Vastan mine (early Eocene, India; Rose et al., 2007; Rose et al., 2009), it is smaller than *A. marandati* (Fig. 6). In addition, *Asiadapis* lacks the first lower premolar, whereas *A. marandati* has a small P_1_. The P_4_ of *A. marandati* has distinct paraconid and metaconid, whereas theses cuspids are absent in some specimens of *A. cambayensis*. The paraconid of the M_1_ is better developed in *A. marandati*. Furthermore, some M_2_ and M_3_ of *A. cambayensis* show a paraconid, which is absent in *A. marandati.* Besides, the talonid basin has a rounded outline in the lower molars of *A. marandati*, whereas in *A. cambayensis* the talonid basin is more elongated mesiodistally*.* Regarding the upper teeth, *A. marandati* shows more developed hypocone, paraconule and metaconule, especially in the M^2^. Furthermore, *A. marandati* displays slightly wrinkled enamel on the M^2^ and M^3^, which is smooth in *A. cambayensis.*

## Results of the Phylogenetic Analyses

**Figure 7:**
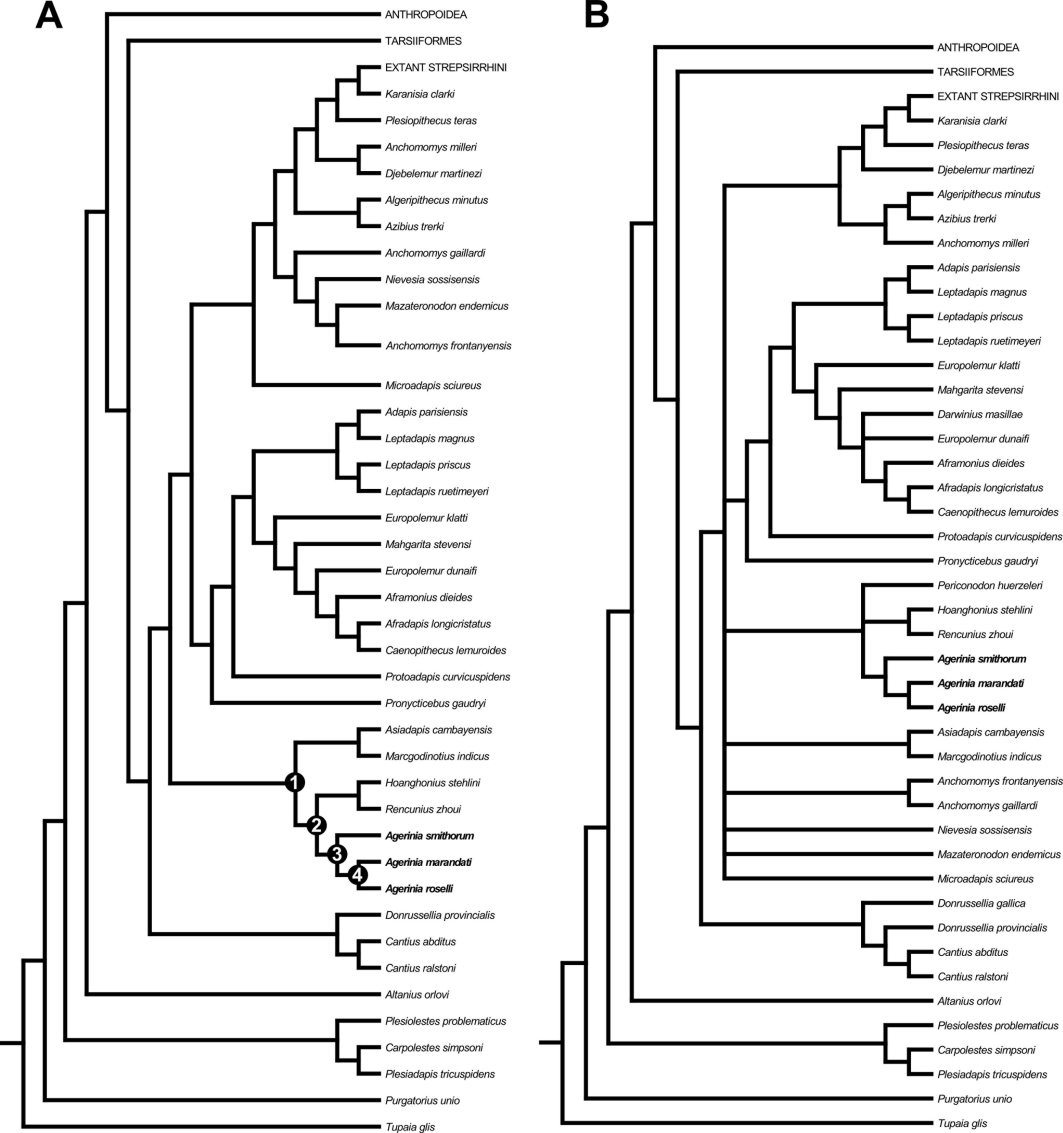
Strict consensus trees derived from parsimony analyses of the 391 character matrix. (A) original data matrix using 109 taxa, strict consensus of 3 equally parsimonious trees (tree length (TL) = 4292.5, consistency index (CI) = 0.163, retention index (RI) = 0.571) recovered by 5,000 heuristic search replicates in PAUP 4.10b10. (B) data matrix with 112 taxa (addition of *Donrussellia gallica, Periconodon huerzeleri* and *Darwinius masillae*), strict consensus of 103 equally parsimonious trees (tree length (TL) = 4,345, consistency index (CI) = 0.162, retention index (RI) = 0.567) recovered by 5,000 heuristic search replicates in PAUP 4.10b10. Unambiguous synapomorphies supporting nodes 1, 2, 3 and 4 are provided in Data S3.

The two developed phylogenetic analyses agree in placing all the species of *Agerinia* together in the same clade. Besides, both analyses place *A. smithorum* as the most primitive of the three species of the genus (Fig. 7). However, both analyses present different results. On the one hand, the first analysis (Fig. 7A), performed taking into account 109 taxa, places the genus *Agerinia* as closely related to the sivaladapids *Hoanghonius* and *Rencunius* and, to a lesser extent, the asiadapines *Asiadapis* and *Marcgodinotius*. In this analysis, the clade formed by *Agerinia,* sivaladapids and asiadapines would not be nested within a monophyletic Adapiformes. These results have been obtained in previous analyses (see Marigó et al., 2016).

Three unambiguous synapomorphies (see Data S3) support the placement of asiadapines as the sister group of the clade formed by *Agerinia* and sivaladapids on the strict consensus tree, and all three synapomorphies are related to dental features. Four unambiguous synapomorphies support the clade formed by *Agerinia* and sivaladapids, and all four are also related to dental features.

On the other hand, when performing the same analysis but taking into account three more taxa (*Donrussellia gallica, Periconodon huerzeleri* and *Darwinius masillae*), the placement of many taxa remains unresolved (Fig. 7B). For instance, the clade formed by *Agerinia*, sivaladapids and asiadapines in the previous analysis, in this second analysis is formed by *Agerinia*, sivaladapids and *Periconodon*, whereas asiadapines are in polytomy with anchomomyins (fully resolved in the previous analysis), as well as “adapiforms” and stem and crown strepsirrhines.

The addition of these three adapiform taxa, which present many characters coded as missing or *unknown*, results in unresolved conditions because only those groups that are found in all trees are included in the consensus tree (Rohlf, 1982). Thus, we conclude that, even if some of these taxa have traditionally been suggested as being closely related to *Agerinia*, their inclusion in phylogenetic analyses may not be the best option until these taxa are further studied or more material is recovered.

## Discussion

The new material presented here represents the most complete sample of the genus *Agerinia* known to date and includes some dental elements previously undescribed, such as the upper molars, the upper and lower deciduous fourth premolars and the P_2_. Besides, the clear differences observed between this material and the previously described species *A. roselli* and *A. smithorum* have allowed erecting the new species *Agerinia marandati.*

The only mandibular fragment of *A. marandati* shows an oblique protruding stripe on the lingual surface of the mandible that suggests an unfused mandible. This feature is observed for the first time in the genus (because the mandibles of *A. smithorum* and *A. roselli* do not preserve this part). Several early Eocene notharctids, such as the genus *Cantius* or the asiadapines *Marcgodinotius* and *Asiadapis*, also display unfused mandibles (Rose et al., 2007; Rose et al., 2009; Godinot, 2015; Smith et al., 2016). Therefore, this feature observed in *Agerinia* could be interpreted as a primitive trait within Notharctidae.

It is also worth noting that *A. marandati* is the only formally described species of *Agerinia* preserving the upper molars. The lack of pericone in the specimens from Masia de l’Hereuet gives further support to the distinction between *Agerinia* and *Periconodon*, this latter genus being mainly characterized by a well-developed pericone.

Besides, the sample from Masia de l’Hereuet allows the evolution of several traits in the three known species of *Agerinia* to be observed. *Agerinia marandati* is similar in size to A. *smithorum* and *A. roselli* (Fig. 6), but displays some morphological differences that have allowed the description of a new species. Indeed, *A. marandati* shows a set of intermediate features between A. *smithorum* and *A. roselli*, suggesting that it represents a transitional step in the evolution of this lineage. Therefore the description of this new species reinforces the idea of the ancestor-descendant relationship between the other mentioned species, as proposed by Femenias-Gual et al. (2016b). These trends are supported by the stratigraphic position of the studied localities: as explained, the type locality of *A. marandati*, Masia de l’Hereuet, is situated in an upper stratigraphic position with respect to Casa Retjo-1, type-locality of *A. smithorum*.

**Table 2:** Comparison of the main morphological traits observed in the three known species of *Agerinia*.

	*A. smithorum*	*A. marandati*	*A. roselli*
P_1_ root			
Position in the mandible	Central	Central	Shifted buccally
Section of the root	Circular	Slightly mesiodistally compressed	Very mesiodistally compressed
P_2_ roots	Two	One	One
P_4_ molarization			
Paraconid	Absent	Distinct	Distinct
Metaconid	Distinct but small	Distinct	Well-differentiated
Hypoconid	Absent	Distinct	Distinct
Entoconid	Absent	Absent	Distinct
M_1_ paraconid	Well-developed	Well-developed	Tiny
M_2_ paraconid	Tiny	Absent	Absent
Position of the mental foramina:			
Distal foramen	Below the P_4_ mesial root	Below the P_4_ mesial root	Below the P_3_ distal root
Intermediate foramen	Below the P_3_ mesial root	Below the P_3_ mesial root	Below the P_2_ root
Mesial foramen	Below the P_1_ root	Between P_1_ and canine	Not observable

**Figure 8:**
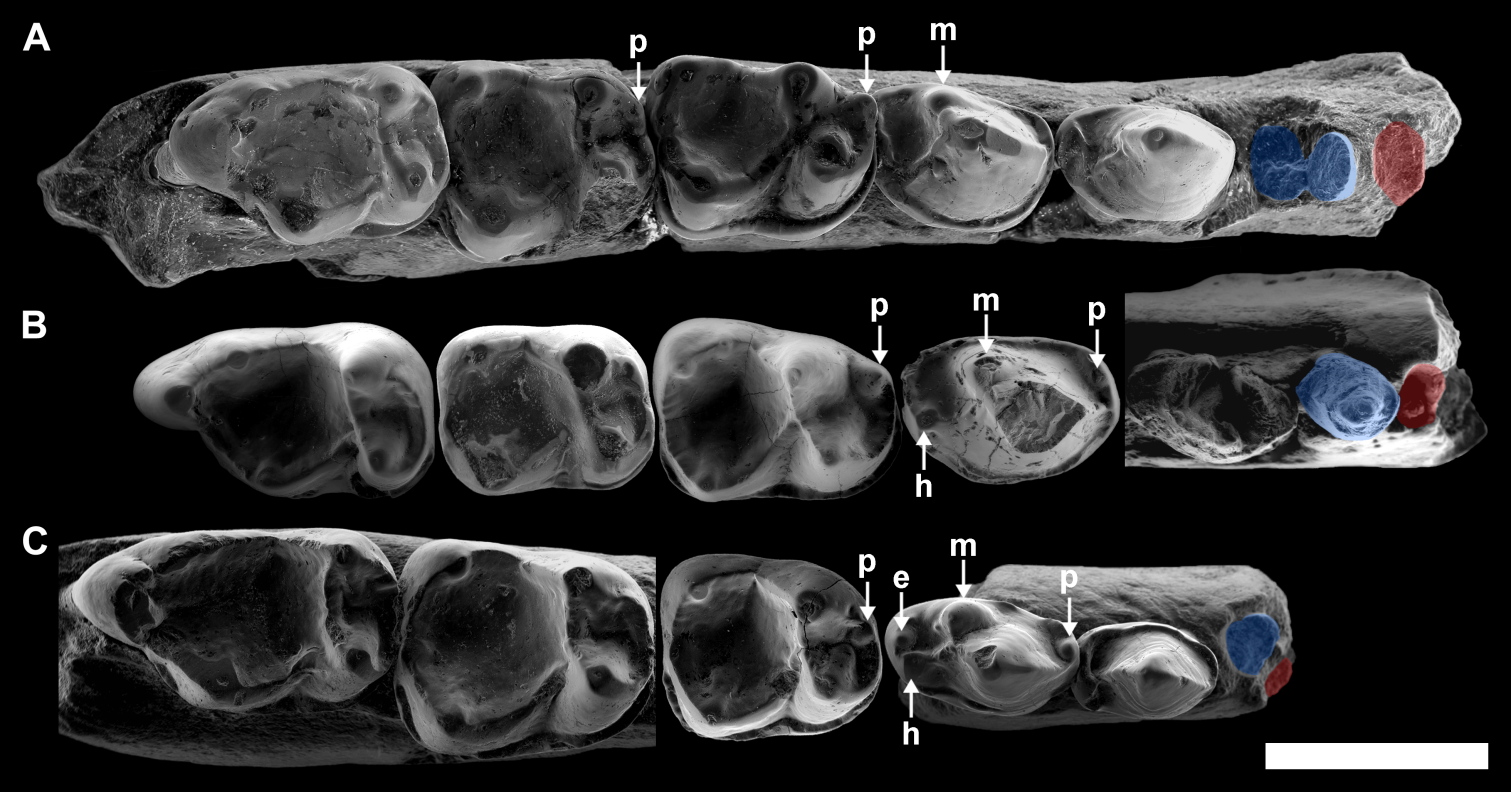
Comparison among the species of the genus *Agerinia.* (A) *A. smithorum* from Casa Retjo-1: IPS-84291, holotype, right mandible with alveoli of the canine and P_1_, roots of the P_2_ and all teeth from P_3_ to M_3_. (B) *A. marandati* from Masia de l’Hereuet: IPS-82807, right mandible fragment with alveoli of the canine and P_1_, and teeth from P_2_ to P_4_ (the P_4_ of this specimen is not shown because of its bad preservation); IPS-82806, left P_4_ (reversed); IPS-82801, holotype, left M_1_ (reversed); IPS-82805, left M_2_ (reversed); IPS-82803, left M_3_ (reversed). (C) *A. roselli* from Les Saleres: IPS-2543, left mandible fragment with roots of the P_1_ and P_2_, and teeth from P_3_ to P_4_ (reversed); IPS-82793, right M_1_; IPS-1981, holotype, left mandible fragment with M_2_ and M_3_ (reversed). White arrows indicate the cuspids: **p**, paraconid; **m**, metaconid; **h**, hypoconid; **e**, entoconid. The root of the P_1_ is highlighted in red and those of the P_2_, in blue. All specimens are represented in occlusal view. Scale bar represents 3 mm.

Table 2 summarizes the main morphological traits in the three described species of *Agerinia*. One of the most remarkable differences is the arrangement and the number of roots of the lower premolars. Although P_1_ is not preserved in any species of the genus, the size and the position of its root changes from *A. smithorum* to *A. roselli* (Fig. 8). In *A. smithorum,* the root of the P_1_ has a circular section and is located centrally in the mesiodistal axis of the mandible. In *A. marandati,* the root of the P_1_ is also centred, but more compressed mesiodistally than in *A. smithorum*. Finally, in *A. roselli* the root of the P_1_ is small and markedly displaced towards the buccal part of the mandible. The existence of two roots mesial to the P_3_ in *A. roselli* led Femenias-Gual et al. (2016a) to consider two different possibilities: the existence of single-rooted P_1_ and P_2_, or the presence of a double-rooted P_2_ extremely oblique to the mandible axis. This latter disposition of the roots of the P_2_ has been observed in some specimens of *Marcgodinotius indicus* (Rose et al., 2007; Rose et al., 2009; Smith et al., 2016). However, the mesial-most root of the mandible of *A. roselli* from Les Saleres is much more shifted buccally than in the case of *Marcgodinotius* so, if the two roots of *A. roselli* would correspond to a double-rooted P_2_, this premolar would be virtually transversal to the longitudinal axis of the mandible (while it is centrally placed and mesiodistally oriented in both *A. smithorum* and *A. marandati*). For this reason, Femenias-Gual et al. (2016a) considered these roots to correspond to single-rooted P_1_ and P_2_. This interpretation is consistent with the rest of features observed in the three known species of *Agerinia*. The material from Les Saleres displays more derived features than *A. marandati* (such as the more molarized P_4_ or more reduced paraconid in the M_1_), which agrees with a reduction and buccal displacement of the P_1_. On the contrary, it seems less plausible that a double-rooted P_2_ evolves from the single-rooted P_2_ of *A. marandati* (Fig. 8).

Despite the small sample size, the observed differences in the P_1_ and P_2_ of these three species seem to indicate a trend towards the reduction of the lower premolars from *A. smithorum* to *A. roselli*, involving the decrease in size of these teeth, the reduction of the number of roots of the P_2_ and the displacement of the P_1_. Such a change has been observed in other primate lineages as *Teilhardina*, in which the P_1_ is progressively reduced and displaced toward the buccal side from older to younger species (Smith, Rose & Gingerich, 2006), being even lost in the youngest species, *T. americana*.

A change in the morphology of the P_4_ can be also observed from *A. smithorum* to *A. roselli*, towards an increase in number and a better development of the cuspids (Fig. 8). *Agerinia smithorum* shows distinct protoconid and metaconid; *Agerinia marandati* shows, in addition, well-developed paraconid and hypoconid; finally, *A. roselli* also shows a entoconid as developed as the paraconid and hypoconid. Furthermore, the metaconid is progressively better differentiated from the protoconid from *A. smithorum* to *A. roselli.*

Regarding the lower molars, in *A. smithorum* the M_1_ has a well-developed paraconid and an open trigonid basin, and the M_2_ shows a tiny paraconid and a closed trigonid basin. *Agerinia marandati* has a similar morphology of the M_1_, with a large paraconid and an open trigonid basin, but lacks the paraconid on the M_2_. On the contrary, *A. roselli* only displays a tiny paraconid on the M_1_, in which the trigonid basin is closed. It is widely known that the lower molar paraconid tends to be reduced during the evolution of different primate lineages (Ankel-Simons, 2007; Godinot, 2015); for example in the genera *Pseudoloris* and *Necrolemur* the size of the paraconid decreases with time, changing from a distinct cuspid to a crest or even becoming completely absent (Minwer-Barakat, Marigó & Moyà-Solà, 2010; Minwer-Barakat, Marigó & Moyà-Solà, 2015; Minwer-Barakat et al., 2015). The same trend can be identified in the species *A. smithorum, A. marandati* and *A. roselli* (Fig. 8).

**Figure 9:**
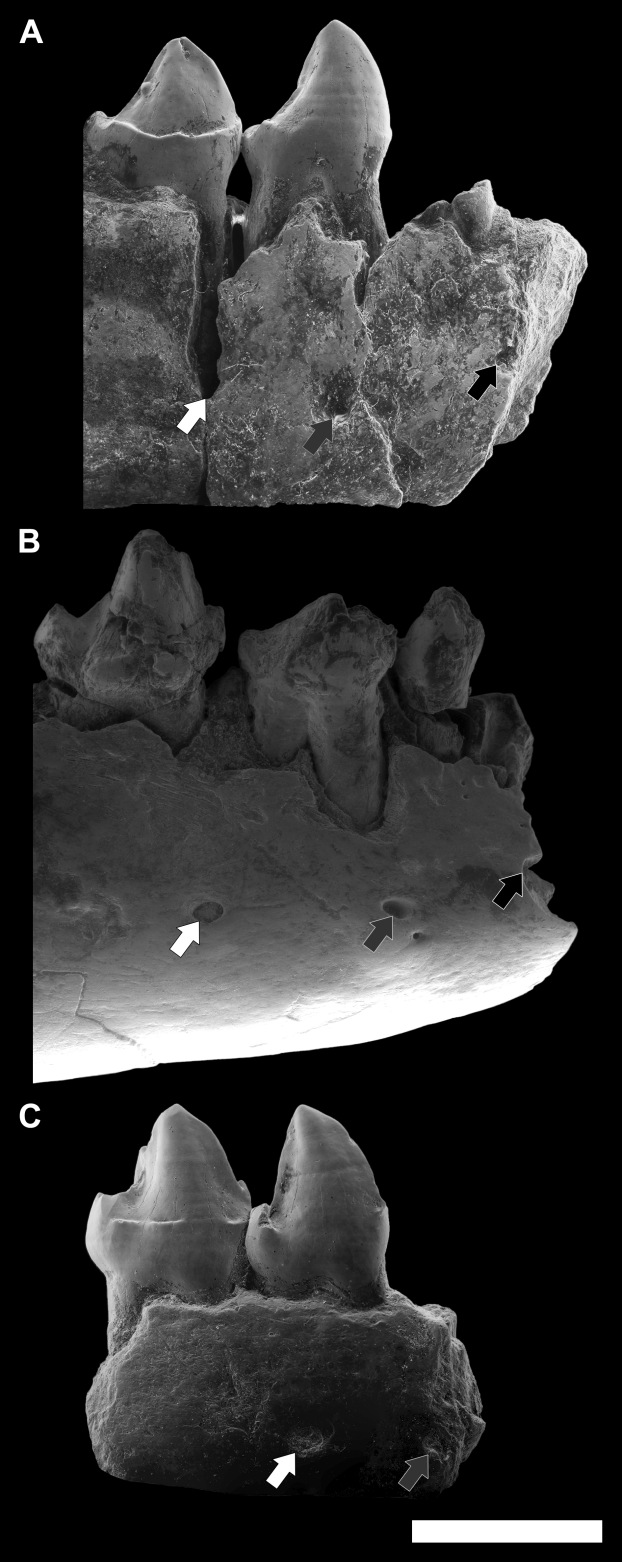
Comparison among the species of the genus *Agerinia*. (A) *A. smithorum* from Casa Retjo-1: IPS-84291, holotype, right mandible with alveoli of the canine and P_1_, roots of the P_2_ and all teeth from P_3_ to M_3_ (this figure only shows teeth from P_3_ to P_4_). (B) *A. marandati* from Masia de l’Hereuet: IPS-82807, right mandible fragment with alveoli of the canine and P_1_, and teeth from P_2_ to P_4_. (C) *A. roselli* from Les Saleres: IPS-2543, left mandible fragment with roots of the P_1_ and P_2_, and teeth from P_3_ to P_4_ (reversed). White arrows indicate the position of the distal-most mental foramen; grey arrows indicate the position of the intermediate mental foramen; black arrows indicate the position of the mesial-most mental foramen. All specimens are represented in buccal view. Scale bar represents 3 mm.

Finally, another remarkable trait that changes in the three species of *Agerinia* is the position of the mental foramina, which shift mesially from *A. smithorum* to *A. roselli* (Fig. 9). In *A. smithorum* and *A. marandati,* the distal-most mental foramen is placed under the mesial root of the P_4_, whereas it is placed at the level of the distal root of the P_3_ in *A. roselli*. The intermediate mental foramen is placed at the level of the mesial root of the P_3_ in *A. smithorum* and *A. marandati* (slightly more mesial in the latter), and at the level of the P_2_ root in *A. roselli*. Finally, the mesial-most mental foramen is located at the level of the P_1_ root in *A. smithorum* and between the P_1_ and the canine alveoli in *A. smithorum* (this foramen cannot be observed in *A. roselli*, since the mandible is broken at its mesial part).

All these considerations are supported by the phylogenetic analyses developed in this work. Both analyses place the three known species of *Agerinia* in the same clade, being *A. smithorum* the most primitive species. These results clearly reinforce the hypothesis of a single evolutive lineage, in which *A. marandati* represents a transitional step in the evolution between *A. smithorum* and *A. roselli*. Regarding the relationships of *Agerinia* with other primates, both analyses group this genus with the sivaladapids *Rencunius* and *Hoanghonius*. In the first analysis (Fig. 7A), *Agerinia* and sivaladapids are closely related to the asiadapines *Marcgodinotius* and *Asiadapis*. In our analyses, “Adapiformes” is not a monophyletic group, as has been previously hypothesized by several authors (Beard et al., 1988; Seiffert et al., 2009; Seiffert et al., 2010; Marigó, Minwer-Barakat & Moyà-Solà, 2011; Marigó, Minwer-Barakat & Moyà-Solà, 2013; Marigó et al., 2016 among others). These results support the idea that the phylogeny of “adapiforms” may be more complicated than previously thought, and highlights this controversy as still one of the most debated topics in paleoprimatology.

To sum up, there are several morphological traits that change progressively from *A. smithorum* to *A. marandati* and finally to *A. roselli*: the number of roots and the position of the first and second lower premolars, the degree of molarization of the P_4_, the development of the paraconid in the M_1_ and M_2_, and the position of the mental foramina. The observed trends suggest that these species constitute a single anagenetic lineage that evolved during the early Eocene in the Iberian Peninsula, in which *A. marandati* represents an intermediate stage of evolution between *A. smithorum* and *A. roselli*.

## Conclusions

Here we present the most complete sample of the genus *Agerinia* described to date, coming from Masia de l’Hereuet (NE Spain). This material displays clear differences with the other species of *Agerinia,* allowing the erection of the new species *Agerinia marandati,* which is characterized by single rooted P_1_ and P_2_; P_4_ with distinct paraconid, protoconid, metaconid and hypoconid; paraconid well-developed in the M_1_ and absent in the M_2_ and M_3_; upper molars with the paraconule more developed than the metaconule and without pericone; M^1^ and M^2^ with a distinct hypocone and well-developed hypoparacrista and preparaconule crista. Moreover, this work describes for the first time the upper and lower fourth deciduous premolars of the genus, as well as some traits of the mandible. The material from Masia de l’Hereuet also includes several upper molars, which were not known for the other species of *Agerinia*. The description of the upper molars reinforces the distinction between the genera *Agerinia* and *Periconodon*, which has been a controversial issue in the past.

*Agerinia marandati* displays intermediate morphological characters between *A. roselli* and *A. smithorum*. A trend has been observed from *A. smithorum* to *A. roselli* for a set of features, including the reduction of the mesial lower premolars, the molarization of P_4_, the reduction of paraconid in the lower molars and the mesial displacement of the mental foramina. These trends allow for the interpretation that the three species of *Agerinia* known from the early Eocene of Europe, *A. smithorum, A. marandati* and *A. roselli*, are integrated in a single evolutionary lineage. Masia de l’Hereuet is situated stratigraphically above Casa Retjo-1 (type locality of *A. smithorum*), which indicates a younger age for *A. marandati* and is therefore consistent with the interpretation of these two species as ancestor and descendant. Finally, the phylogenetic analyses developed in this work support the hypothesis of a single clade including the three species of *Agerinia,* and indicate that *Agerinia smithorum* is the most primitive species of the genus.

##  Supplemental Information

10.7717/peerj.3239/supp-1 Data S1 Character-taxon matrix used for the phylogenetic analyses

10.7717/peerj.3239/supp-2 Data S2 Constraint tree used for the phylogenetic analyses

10.7717/peerj.3239/supp-3 Data S3 List of synapomorphies of different nodes specified in Fig. 9

## Additional Information and Declarations

### Data Availability

The following information was supplied regarding data availability: The raw data has been supplied as Supplemental Files.

### New Species Registration

The following information was supplied regarding the registration of a newly described species: Genus name: urn:lsid:zoobank.org:act:1EC6B7E1-DA78-4AFE-B24E-E4D56F0D861D Species name: urn:lsid:zoobank.org:act:02E82A3B-58B0-4FB8-AE2C-64E1517A0F24 Publication LSID: urn:lsid:zoobank.org:pub:729814E7-5509-48C1-9FF0-84D3E515D909.
