# Supplementary material for: Agerinia marandati sp. nov., a new early Eocene primate from the Iberian Peninsula, sheds new light on the evolution of the genus Agerinia
Source: PeerJ. 2017 Apr 27;5:e3239. doi: 10.7717/peerj.3239 (PMC5410143; doi:10.7717/peerj.3239)
Supplement: Data S3 [file peerj-05-3239-s003.docx]

Node 1:

68: m1 cristid obliqua terminus 6 ==> 2

72: m1-2 hypocristid development 4 ==> 3

94: position of hypoconulid on m3 0 ==> 1

Node 2:

60: m1 area 4 ==> 6

66: m1 trigonid height (ratio of trigonid height to talonid height) 1 ==> 2

73: m1-2 buccal cingulum development 2 ==> 3

151: M1-2 hypocone position 0 ==> 2

Node 3:

33: p3 entoconid and lingual talonid crest 2 ==> 0

39: mesiodistal position of p4 metaconid with respect to protoconid 0 ==> 1

50: p4 hypocristid shearing development 0==> 2

52: lower premolar inflation 0 ==> 2

66: m1 trigonid height (ratio of trigonid height to talonid height) 2 ==> 4

81: position of entoconid relative to hypoconid on m2 2 ==> 3

91: m3 trigonid width (based on relative buccolingual breadths) 2 ==> 4

Node 4:

22: p2 roots/presence 0 ==> 2

35: p3 area/p4 area 5 ==> 4

40: p4 paraconid size 4 ==> 3

64: m1 premetacristid 0 ==> 1

70: m1 hypoconulid size 4 ==> 6

83: m2 hypoconulid size 4 ==> 5
